# Genome-Wide Association Study on Root System Architecture and Identification of Candidate Genes in Wheat (*Triticum aestivum* L.)

**DOI:** 10.3390/ijms23031843

**Published:** 2022-02-06

**Authors:** Jianhui Ma, Dongyang Zhao, Xiaoxiao Tang, Meng Yuan, Daijing Zhang, Mengyuan Xu, Yingze Duan, Haiyue Ren, Qingdong Zeng, Jianhui Wu, Dejun Han, Tian Li, Lina Jiang

**Affiliations:** 1 College of Life Science, Henan Normal University, Xinxiang 453007, China; 2013033@htu.edu.cn (J.M.); zdyzhaoyang@163.com (D.Z.); hnsqtxx@163.com (X.T.); ym140906@163.com (M.Y.); 041160@htu.edu.cn (D.Z.); xumengyuan2022@163.com (M.X.); d1804114003@163.com (Y.D.); rhy20010621@163.com (H.R.); 2 State Key Laboratory of Crop Stress Biology for Arid Areas, Northwest A&F University, Yangling, Xianyang 712100, China; zengqd@nwafu.edu.cn (Q.Z.); wujh@nwafu.edu.cn (J.W.); handj@nwsuaf.edu.cn (D.H.); 3 Key Laboratory of Crop Gene Resources and Germplasm Enhancement, Ministry of Agriculture, Institute of Crop Sciences, Chinese Academy of Agricultural Sciences, Beijing 100081, China

**Keywords:** *Triticum aestivum* L., root system architecture, genome-wide association study, quantitative trait loci, candidate genes

## Abstract

The root tissues play important roles in water and nutrient acquisition, environmental adaptation, and plant development. In this study, a diversity panel of 388 wheat accessions was collected to investigate nine root system architecture (RSA) traits at the three-leaf stage under two growing environments: outdoor pot culture (OPC) and indoor pot culture (IPC). Phenotypic analysis revealed that root development was faster under OPC than that under IPC and a significant correlation was observed between the nine RSA traits. The 660K single-nucleotide polymorphism (SNP) chip was used for a genome-wide association study (GWAS). Significant SNPs with a threshold of −log_10_ (*p*-value) ≥ 4 were considered. Thus, 36 quantitative trait loci (QTLs), including 13 QTL clusters that were associated with more than one trait, were detected, and 31 QTLs were first identified. The QTL clusters on chromosomes 3D and 5B were associated with four and five RSA traits, respectively. Two candidate genes, *TraesCS2A01G516200* and *TraesCS7B01G036900*, were found to be associated with more than one RSA trait using haplotype analysis, and preferentially expressed in the root tissues. These favourable alleles for RSA traits identified in this study may be useful to optimise the root system in wheat.

## 1. Introduction

Bread wheat (*Triticum aestivum* L.) is one of the most important staple crops worldwide. The cultivated area of wheat is approximately 217 million hectares, annually producing approximately 765 million tonnes [1]. However, the demand for wheat continues to increase owing to a rapid increase in population. According to the speculation by the Food and Agriculture Organisation of the United Nations, approximately 840 million tonnes of wheat will be required annually by 2050 [2]. However, wheat production is facing serious challenges with the increasing global climate change in recent years [3,4]. Wheat breeding for high yield and stress tolerance will remain the main strategy for a long time. Roots, the underground parts of plants, absorb nearly all of the nutrients required for plant development and are primarily exposed to osmotic stress [5]. Optimal RSA improves the wheat yield and stress tolerance [6,7]. Therefore, it is important to study the genetic mechanisms of crop RSA traits to optimise the root system for further breeding.

RSA traits include root length, root surface area, root diameter, root tip number, and root volume [8]. RSA traits play important roles in determining crop development [9,10], yield formation [11,12], and stress tolerance [13]. For marker-assisted selection and key gene identification, the QTLs for RSA traits were explored in different crops [14,15]. Zhang et al. [16] analysed the seminal root length in rice using a recombinant inbred line (RIL) population of 150 lines under different water supply conditions, and identified two QTLs that explained 10% and 11% of phenotypic variation. Li et al. [17] identified 46 environment-specific QTLs for the crown root traits in maize, among which 12 QTLs were detected in multiple environments. Wu et al. [18] identified two major QTLs of swollen roots in turnips, providing a new idea regarding the molecular mechanism underlying storage root formation. In addition, Ren et al. [19] collated the QTLs for RSA and other traits, and found overlaps between QTLs for the root traits and those for nutrient uptake and productivity in many crop species, suggesting that RSA traits are closely related to crop development. These researches provided abundant information for further marker-assisted selection. Steele et al. [20] constructed introgressed lines using the previously reported root QTLs, which significantly improved the rice yield. Therefore, studying RSA traits is of great significance.

Bread wheat is consumed by 30–40% of humans [1]. Many researchers have paid attention to wheat root tissues. Manschadi et al. [10] analysed drought adaptation using wheat seedlings, and revealed that the root tissues are more closely related to drought tolerance than the aboveground tissues. Li et al. [21] found that deep roots, optimal root length density and xylem diameter, and increased root surface area contribute to drought tolerance. To elucidate the mechanisms of root development, many studies on QTL mapping of RSA traits have been performed in wheat. Liu et al. [22] analysed RSA traits using a diverse panel of 165 wheat cultivars, and identified 28 and 4 QTLs in hydroponic and field environments, respectively. Alemu et al. [23] analysed RSA traits using 192 Ethiopian durum wheat accessions, and identified 4 major (–log_10_*p* ≥ 4) and 34 nominal (–log_10_*p* ≥ 3.5) QTLs. Fan et al. [24] analysed the RSA traits in the seedlings of 188 RILs subjected to different nitrogen concentrations, and detected 53 QTLs responsible for RSA traits. In total, 26 published reports performed QTL exploration for RSA traits [5,19,22,23,24,25,26,27,28,29,30,31,32,33,34,35,36,37,38,39,40,41,42,43,44,45], which provided ample information on RSA traits in wheat.

The wheat genome is enormous and complex. Research on the quantitative traits in wheat lags behind the research on other crops [28]. Especially for the RSA traits, each trait is controlled by multiple genes. Although researchers identified many QTLs for RSA traits, a few genes were functionally verified to regulate root development using the forward genetic approaches in wheat [46,47]. The mechanism for the RSA traits remains unclear, and more loci need to be explored in wheat. For the completion of genome sequencing, many types of SNP chips were developed, and the 660K SNP chip was considered to be the optimal chip for GWAS in wheat [48]. In this study, a diversity panel of 388 wheat accessions, based on pre-diversity assessment, was used to analyse RSA traits at the seedling stage under two environments. The 660K SNP chip was used for GWAS. We expect to identify vital QTLs and candidate genes to improve RSA.

## 2. Results

### 2.1. Population Structure and Linkage Disequilibrium (LD) Decay Analysis

**Figure 1:**
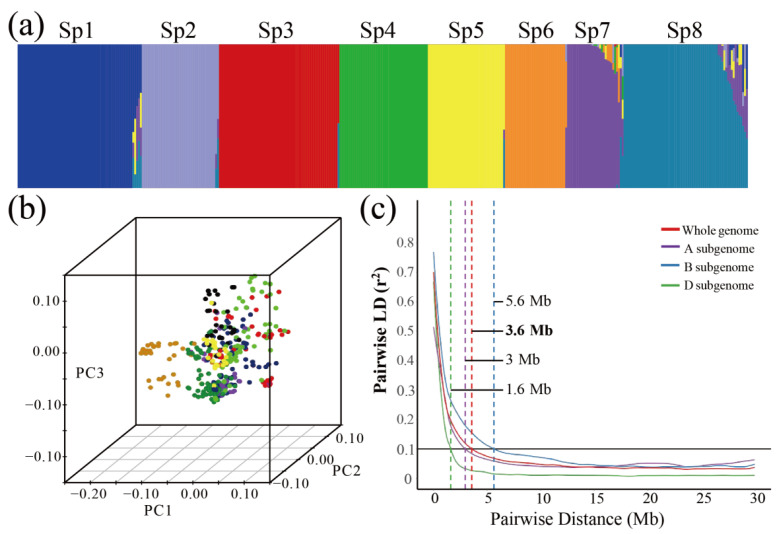
Population structure and LD analysis of the wheat panel. (**a**) Subpopulations inferred by *K*-means structure analysis. (**b**) Principal component analysis of all wheat accessions. (**c**) Genome-wide average LD decay over physical distance. Pair-wise single-nucleotide polyLD (*r*^2^) values based on the physical positions from the IWGSC RefSeq v.1.0 reference genome are plotted as a function of mapping distance (Mb) between markers. The different-coloured curves represent LD decay fits for sub-genomes A (purple), B (blue), D (green), and the whole genome (red). The thick horizontal black line represents the population-specific critical *r*^2^ value (0.1) above which LD may be due to linkage.

The population structure was determined using STRUCTURE V2.3.4 according to the ∆*K* method of Bayesian clustering. We determined the slope breakpoint at *K* = 8. Thus, the panel used in this study was divided into eight subpopulations (Sp1-Sp8, Figure 1a, Table S1). Sp1 comprised 11 exotic varieties, 6 landraces, and 49 local cultivars. Sp2 consisted of 7 exotic varieties, 4 landraces, and 29 local cultivars. Sp3 comprised 5 exotic varieties, 1 landrace, and 58 local cultivars. Sp4 contained 13 exotic varieties, 15 landraces, and 19 local cultivars. Sp5 was composed of 10 exotic varieties, 3 landraces, and 28 local cultivars. Sp7 consisted of 1 exotic variety, 1 landrace, and 28 local cultivars. Sp8 contained 19 exotic varieties, 7 landraces, and 40 local cultivars (Table 1, Figure 1b).

We estimated the degree of LD using 161,801, 184,658, and 58,479 SNP markers in the A, B, and D sub-genomes, respectively. LD was analysed based on 717,701,068 pair-wise comparisons of 404,938 SNPs. Pair-wise LD was estimated using the squared allele-frequency correlation (*r*^2^). A plot of the LD estimates (*r*^2^) as a function of physical distance (Mb) indicated clear LD decay with increasing physical distance (Figure 1c). A comparison of LD between the sub-genomes and chromosomes showed variances in the LD decay. Overall, the average LD decay distance in the whole genome was approximately 3.6 Mb. LD decayed faster in the D genome (1.6 Mb) than in the A (3 Mb) and B (5.6 Mb) genomes (Figure 1c). The faster LD decay in the D genome is compatible with the evolutionary history of wheat.

### 2.2. Phenotypic Variation of the RSA Traits

**Figure 2:**
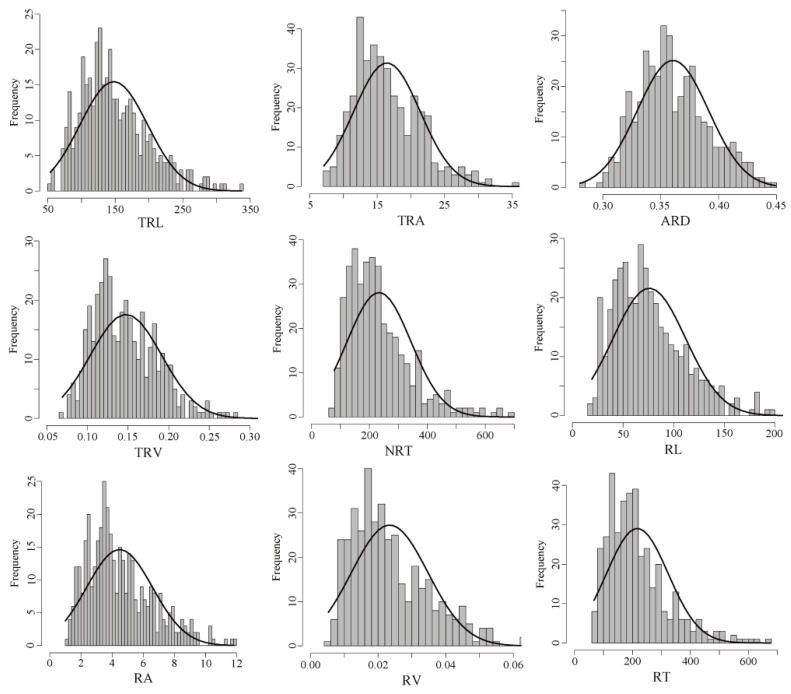
Distribution frequency of the RSA traits of 388 wheat accessions under OPC. The black curve represents the density curve.

A total of 4656 (388 wheat accessions × 6 replications × 2 environments) seedling roots were collected and observed for the nine RSA traits namely, total root length (TRL), total root surface area (TRA), average root diameter (ARD), total root volume (TRV), number of root tips (NRT), root length (RL), root surface area (RA), root volume (RV), and root tips (RT) (Table S1). The frequency distribution of the investigated traits was normal, as indicated by the kurtosis (bk) and skewness (bs) (Figure 2). This means that the traits were quantitative and suitable for GWAS. Phenotypic variation analysis showed that the nine RSA traits exhibited greater variation under OPC than under IPC, indicated by the coefficient of variation (Table 2). In addition, the nine RSA traits were significantly higher under OPC than under IPC (*p* ≤ 0.001). In other words, the root development was faster under OPC than that under IPC. The broad-sense heritability (*H*^2^) of the nine RSA traits ranged from 66.78% to 77.83%, indicating that the variations observed in these RSA traits were mainly regulated by genotype.

### 2.3. Correlations between RSA Traits

**Figure 3:**
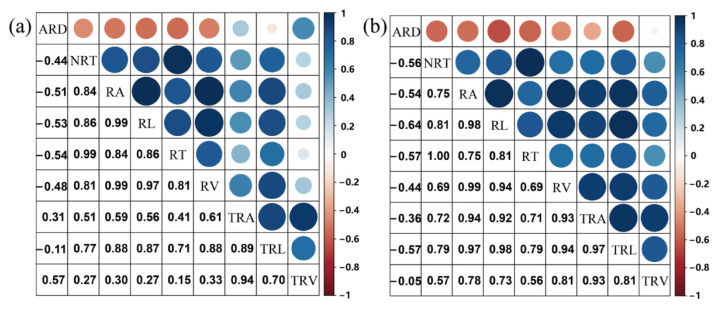
Correlation analysis of the nine RSA traits in 388 wheat accessions under OPC (**a**) and IPC (**b**). Intensities of the blue and red colours indicate the degree of positive and negative correlations, respectively. Circle size indicates low to high significance.

The relationships of the nine RSA traits between the two environments were analysed. The results showed that each RSA trait showed a significant correlation (*p* ≤ 0.001) between the two environments. This means that these traits were mainly regulated by genotype. The relationships between the nine traits were calculated (Figure 3). The results showed that significant positive correlations were observed between eight RSA traits, except for ARD. ARD showed a significant negative correlation with NRT, RL, RA, RV, and RT in the two environments. The correlation coefficients between RL, RA, and RV were higher than 0.94 in the two environments, indicating that these three traits were strongly correlated.

### 2.4. GWAS of RSA Traits

After filtering low-quality SNPs (minor allele frequency < 0.05 and missing data > 0.1), a total of 411,605 polymorphic SNP markers with effective chromosome information were available for GWAS using a univariate mixed linear model of GEMMA (Table S2). Of these, 404,938 SNPs were mapped to 21 wheat chromosomes, with 161,801, 184,658, and 58,479 SNPs in the A, B, and D sub-genomes, respectively. The marker density varied among different chromosomes (Figure S1, Table 3). We discovered that the minimum marker density was 8.1 markers per Mb on chromosome 4D, whereas the maximum was 55.09 markers per Mb on chromosome 3B. Compared with the A and B sub-genomes, the D sub-genome had fewer SNP markers and effective markers, especially chromosome 4D, which contained 1702 SNPs. The markers of known chromosome positions were used to analyse the genetic diversity. We found that there was little difference in the genetic diversity among the three sub-genomes. The mean values of the expected heterozygosity (*He*) and polymorphism information content (PIC) were 0.66–0.74 and 0.27–0.30 among the three sub-genomes, respectively.

We performed a GWAS using the best linear unbiased predictions (BLUPs) of the nine RSA traits. Significant SNPs for each trait were selected with a threshold of –log_10_ (*p*-value) ≥ 3.5 (Table S3, Figure S2). After analysing the data, 133 QTLs evenly distributed on 21 chromosomes were identified for the nine RSA traits, among which 38 were associated with multiple RSA traits. Eight QTLs were overlapped with previously identified QTLs (Table S3).

**Figure 4:**
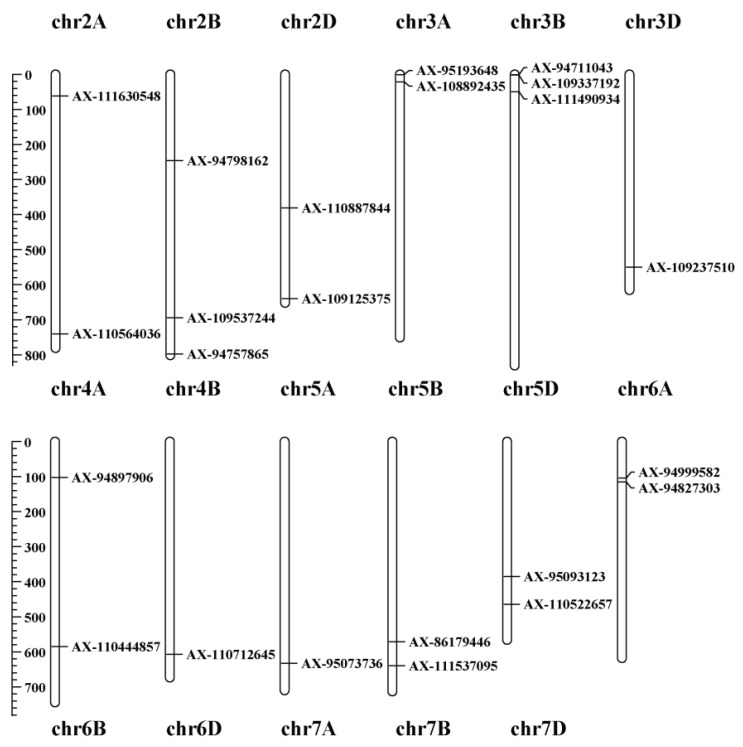

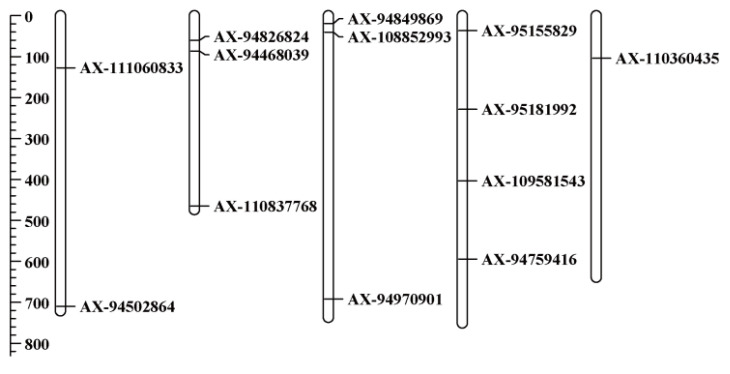
Location of the QTLs for the RSA traits (−log_10_ [*p*-value] ≥ 4).

We focused on significant SNPs with a threshold of –log_10_ (*p*-value) ≥ 4. For convenience, we selected tagged SNPs for each QTL exhibiting the strongest association with RSA traits (Table 4), yielding 36 QTLs on 17 chromosomes (Figure 4), of which 13 were associated with more than one RSA trait. Among these QTLs, five QTLs, mapped on chromosomes 5B, 6B, 6D, 7A, and 7B, were co-localised with those identified in previous studies (Table 4), indicating that our results were reliable.

### 2.5. QTL Clusters for Root System Architecture Traits

QTL clusters refer to the QTLs that were associated with more than one RSA trait. We identified 13 QTL clusters located on chromosomes 2A, 2B, 3A, 3B, 3D, 4A, 5B, 5D, 6B, and 7B. Chromosomes 2A, 3B, and 7B contained two QTL clusters, and chromosomes 2B, 3A, 3D, 4A, 5B, 5D, and 6B harboured a single QTL cluster. One QTL cluster on chromosome 5B (571.23 Mb) was associated with five RSA traits: RL, RA, RV, RT, and NRT. One QTL cluster on chromosome 3D (549.74 Mb) was associated with four RSA traits: RL, RA, RV, and TRL. Two QTL clusters on chromosome 2A (61.28 Mb and 740.01 Mb) and one QTL cluster on chromosome 2B (694.26 Mb) were associated with TRA and TRV. Two QTL clusters on chromosome 7B were associated with TRL and TRA. One QTL cluster on chromosome 3A, two on chromosome 3B, one on chromosome 4A, one on chromosome 5D, and one on chromosome 6B were associated with RT and NRT. Therefore, these QTL clusters showed a pleiotropic effect.

### 2.6. Candidate Genes Associated with RSA

A total of 649 genes were identified in the 13 QTL clusters (Table S4). The genes encoding the bHLH transcription factor, WUSCHEL-RELATED HOMEOBOX 4 (WOX4), ROOT HAIR DEFECTIVE 6 (RHD6), GLABRA 3 (GL3), and ROOTHAIRLESS-LIKE 1 (LRL1), which are closely related to RSA traits, were found in the QTL clusters. We analysed the significant SNPs in thirteen QTL clusters, and identified nine genes with non-synonymous polymorphisms across the wheat panel (Table S5). The phenotypic variation related to each haplotype of the above nine genes was characterised, and the phenotypic differences between the haplotypes of two genes, *TraesCS2A01G516200* with the SNP AX-110564036 (GG/AA) and *TraesCS7B01G036900* with the SNP AX-112287343 (CC/TT), were significant (*p* ≤ 0.05) in more than one RSA trait.

**Figure 5:**
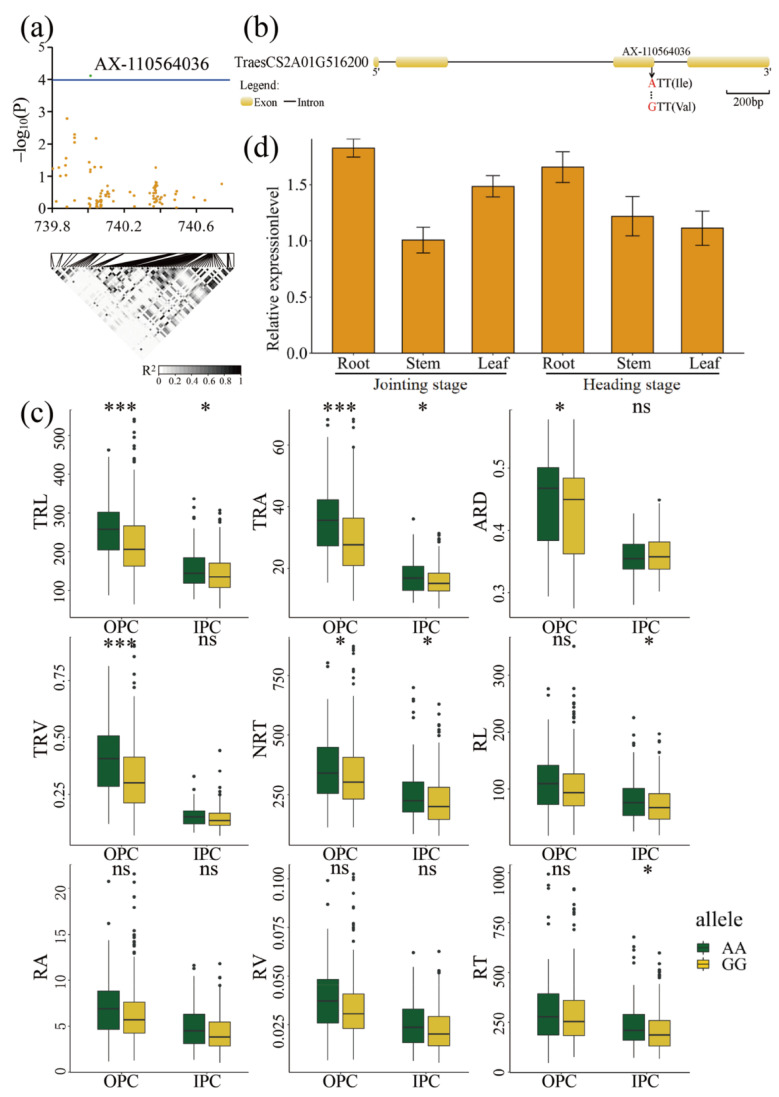
GWAS results of the nine RSA traits and identification of candidate gene for the SNP AX-110564036 on chromosome 2A. (**a**) Manhattan plot for the candidate region on chromosome 2A. The green dot represents the significant SNP AX-110564036. The LD block analysis of the SNPs in this region is shown below. The degree of linkage is represented by the coefficient of *r*^2^. (**b**) Gene structure of *TraesCS2A01G516200* and its non-synonymous SNP. Yellow rectangles and black lines represent exons and introns, respectively. The arrow indicates the position of amino acid variations in *TraesCS2A01G516200*. (**c**) Box plots for RSA traits based on the allele at SNP AX-110564036. Differences between the haplotypes were statistically analysed using the Student’s *t*-test. The upper and lower edges of the box represent the 75th and 25th quantiles, respectively, and the whiskers show the 90th and 10th quantiles; the horizontal solid line represents the average; * Significant at *p* ≤ 0.05, *** Significant at *p* ≤ 0.001. (**d**) Expression profile of *TraesCS2A01G516200* in different tissues, as determined by RT-qPCR. The data are the means ± Sd of *n* = 3. ‘ns’ means ‘not significant’.

*TraesCS2A01G516200* (AX-110564036), mapped to chromosome 2A between 739.8 Mb and 740.8 Mb, encoded a short-chain dehydrogenase/reductase (Figure 5a). AX-110564036 SNP caused an amino acid change from isoleucine (Ile) to valine (Val) at 167 bp in this gene (Figure 5b). The 388 accessions were divided into two haplotypes (GG or AA) according to their genotype at this SNP. We determined that 258 and 93 accessions harboured the GG and AA haplotypes, respectively, whereas the remaining 37 accessions were heterozygous or lacked genotype information. Under the two environments, the accessions carrying the AA allele showed significantly higher values of the RSA traits TRL, TRA, and NRT than those carrying the GG allele (*p* ≤ 0.05), indicating that the AA haplotype had a positive effect on RSA (Figure 5c). RT-qPCR analysis showed that this gene was preferentially expressed in the root tissues at the jointing and heading stages (Figure 5d). These results showed that *TraesCS2A01G516200* plays an important role in the RSA traits of wheat. 

**Figure 6:**
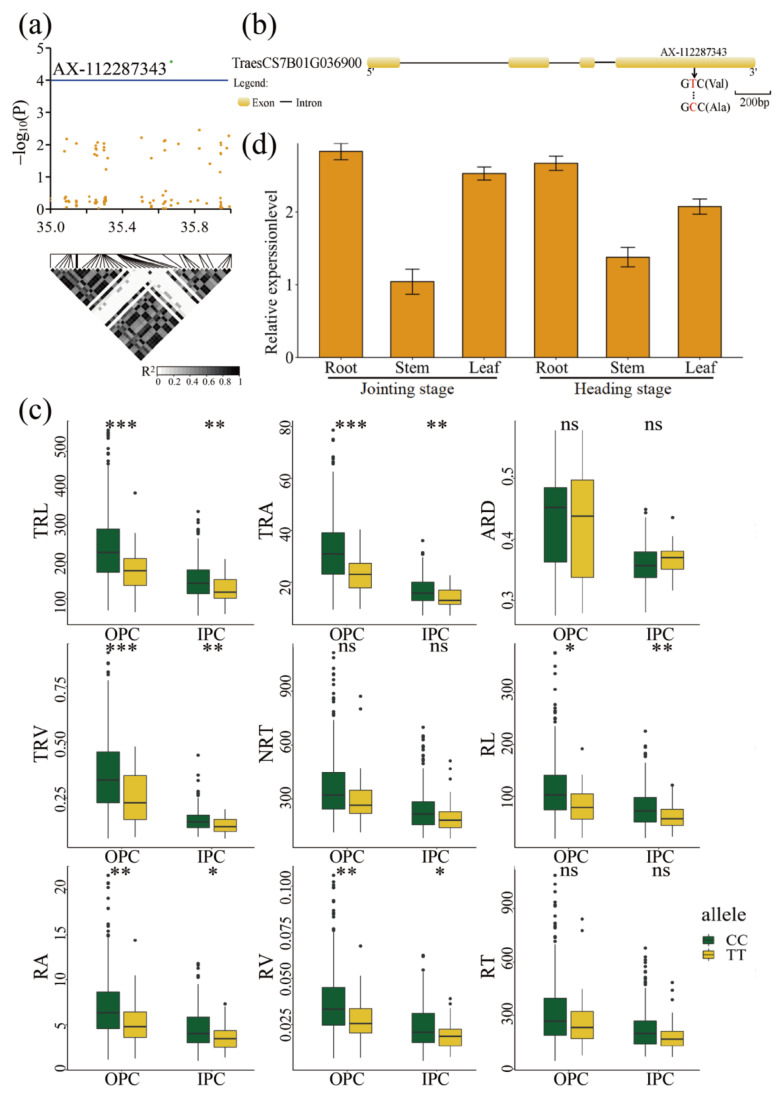
GWAS results of the nine RSA traits and identification of the candidate gene for the SNP AX-112287343 on chromosome 7B. (**a**) Manhattan plot for the candidate region on chromosome 7B. The green dot represents the significant SNP AX-112287343. The LD block analysis of the SNPs in this region is shown below. The degree of linkage is represented by the coefficient of *r*^2^. (**b**) Gene structure of *TraesCS7B01G036900* and its non-synonymous SNP. Yellow rectangles and black lines represent exons and introns, respectively. The arrow indicates the position of amino acid variations in *TraesCS7B01G036900.* (**c**) Box plots for RSA traits based on the allele at SNP AX-112287343. Differences between the haplotypes were statistically analysed using the Student’s *t*-test. The upper and lower edges of the box represent the 75th and 25th quantiles, respectively, and the whiskers show the 90th and 10th quantiles; the horizontal solid line represents the average; * Significant at *p* ≤ 0.05, ** Significant at *p* ≤ 0.01, *** Significant at *p* ≤ 0.001. (**d**) Expression profile of *TraesCS7B01G036900* in different tissues, as determined by RT-qPCR. The data are the means ± Sd of *n* = 3. ‘ns’ means ‘not significant’.

*TraesCS7B01G036900* (AX-112287343), mapped to chromosome 7B between 35 Mb and 36 Mb, encoded a development and cell death domain protein (Figure 6a). AX-112287343 SNP caused an amino acid change from valine (Val) to alanine (Ala) at 396 bp in this gene (Figure 6b). The 388 accessions were divided into two haplotypes (CC or TT) according to their genotype at this SNP. We determined that 348 and 36 accessions harboured the CC and TT haplotypes, respectively, while the remaining four accessions were heterozygous or lacked genotype information. Under the two environments, the accessions with the CC haplotype exhibited markedly higher RSA trait values for TRL, TRA, TRV, RL, RA, and RV than those with the TT haplotype (*p* ≤ 0.05), indicating that the CC haplotype had a positive effect on RSA (Figure 6c). The RT-qPCR analysis indicated that this gene was preferentially expressed in the root tissues at the jointing and heading stages (Figure 6d). These results showed that *TraesCS7B01G036900* plays an important role in the RSA traits of wheat.

## 3. Discussion

### 3.1. Soil Culture and 660K SNp Chip Are Ideal Methods for GWAS of RSA Traits in Wheat 

Bread wheat is a staple crop worldwide, whose RSA traits strongly affect crop development and yield [49]. The spatial distribution of roots affects the absorption of water and nutrients and plant development. Zhu et al. [50] established that the length and number of seminal roots are particularly vital for the uptake of immobile nutrients. Devaiah et al. [51] found that the differences between RSA traits lead to changes in nutrient absorption. In addition, a deeper root system has higher water absorption capacity owing to its better access to soil water [52]. More importantly, White et al. [53] indicated that shoot and root architectures are inherited independently, and could be improved to increase the absorption and utilisation of water and nutrients. Therefore, it is important to analyse the RSA traits and explore the QTLs for these characteristics for further screening of key genes. 

Some studies have identified several QTLs for RSA traits in wheat. To conveniently obtain whole roots, plants were usually cultivated in Hoagland’s nutrient solution, sand, or agarose gel [45,54]. Hydroponics is the most common approach used to obtain the whole roots in wheat [22,31,32,42]. Although the QTLs for RSA traits that were identified through hydroponic cultivation coincided with those for yield and nutrient absorption in wheat [43,44,55], we were not sure whether the phenotype of RSA traits in hydroponics was similar to that in soil cultivation. Therefore, soil cultivation is reliable to investigate RSA traits, although it is time-consuming and difficult. In this study, 388 wheat accessions were cultivated in soil under OPC and IPC, and the whole roots were sampled and cleaned up at the three-leaf stage to obtain the RSA traits for further analysis. The nine RSA traits showed high phenotypic variation at the seeding stage, indicating that the wheat panel used in this study exhibited high genetic diversity. The nine RSA traits under the two environments were significantly correlated, and the correlation between these RSA traits was significant, indicating that some QTLs may contribute to more than one RSA trait. The mean values of these RSA traits under OPC were significantly higher than those under IPC. We found that the soil under the two environments was comparable, and the mean temperature under IPC was higher than that under OPC. However, the root development was faster under OPC than that under IPC. It may be due to the climatic conditions from 15 October 2019 to 9 November 2019 that were optimal for wheat seedlings in Xinxiang (35.29° N, 113.83° E), Henan, China. The broad-sense heritability of the nine RSA traits ranged from 66.78–77.83%, indicating that these traits were suitable for QTL exploration. 

Because of the vastness of genomic data, it is expensive to explore QTLs through genome re-sequencing in wheat. A series of high-density SNP chips, including 820K [56], 50K [57], 55K [58], 90K [33], and 660K chips, were designed and utilised for marker-assisted breeding in bread wheat. The 660K SNP chip contains 229,266 SNPs in the gene or promoter regions of 66,834 genes, representing 63.52% of the annotated genes in wheat. It is an optimal chip for marker-assisted breeding and has been widely used for QTL exploration for quality traits, agronomic traits, and disease resistance in bread wheat [48]. In the present study, the wheat panel was genotyped for GWAS using the 660K SNP chip. It is reliable to screen for significant QTLs associated with RSA traits.

### 3.2. QTL Analysis for RSA Traits 

A substantial number of studies have performed QTL mapping for RSA traits in different plants, of which 26 studies focused on RSA traits in wheat. They identified 410 QTLs for RSA traits (Table 5). In this study, 36 QTLs with a threshold of −log_10_ (*p*-value) ≥ 4 were identified for nine RSA traits. Among these, five QTLs overlapped with previously reported QTLs, and 31 new QTLs were identified. This indicates that these genomic regions for the RSA traits are reliable and provide new information for further studies.

This study identified thirteen QTL clusters associated with more than one RSA trait that were distributed on ten chromosomes, indicating that there were co-localisations among these RSA traits. From the relationship among the nine RSA traits, we found that eight RSA traits showed significant positive correlations, and ARD showed a significant negative correlation with NRT, RL RA, RV, and RT in the two environments. From these results, we found that root traits are closely associated with each other, and some traits could be controlled by the same QTL. This might be because of the pleiotropic effects of the same gene or some RSA traits that might have a common genetic basis.

### 3.3. QTL Analysis Reveals the Key Genes for RSA

Key genes regulate RSA and root development. Therefore, the screening of key genes is important for RSA improvement. Zhang et al. [59] found that WOX4, encoded by AT1G46480, plays a central role in cambium development in *Arabidopsis* roots. Here, we found *TraesCS2A01G514000*, a homologue of *AT1G46480*, in the QTL on chromosome 2A, which was associated with TRA and TRV. Menand et al. [60] found that RHD6, encoded by AT1G66470, was a positive regulator for root hair development. *TraesCS3B01G007700* was found to be homologous with *AT1G66470* and mapped to the QTL of chromosome 3B that was associated with NRT and RT. Bernhardt et al. [61] found that a GL3 protein, encoded by AT5G41315 in *Arabidopsis*, partially regulates root epidermal cell fates. Here, we found that *TraesCS2D01G575600*, a homologue of *AT5G41315*, was located in the QTL of chromosome 2D that was associated with ARD, which might influence root epidermal cell fate. Karas et al. [62] found that LRL1, encoded by AT2G24260, is a partial regulator of root hair development in *Arabidopsis*. Here, we found that *TraesCS7D01G158300*, a homologue of *AT2G24260*, was located in the QTL of chromosome 7D that was associated with ARD. Therefore, these genes were considered candidates for the respective RSA traits. 

In addition, we manually screened the significant SNPs for haplotype analysis, and found that nine genes showed non-synonymous polymorphisms across the wheat panel. Two genes, *TraesCS2A01G516200* (AX-110564036) and *TraesCS7B01G036900* (AX-112287343), have attracted our attention. The different combinations of haplotypes for the two genes showed significant differences for RSA traits. Importantly, the two genes were highly expressed in the root tissues. Functional annotation analysis revealed that *TraesCS2A01G516200* encoded a short-chain dehydrogenase/reductase and *TraesCS7B01G036900* encoded a development and cell death domain protein. These results indicate that these two genes may be vital candidate genes for root architecture. 

## 4. Materials and Methods

### 4.1. Plant Materials and Experimental Treatment

Based on pre-diversity assessment of more than 5000 wheat accessions using KASP markers or 660 K SNP chip, a diversity panel of 388 wheat accessions was collected, including 38 local landraces, 66 exotic cultivars, and 284 cross-derived cultivars in China. The name and origin of each wheat accession are listed in Table S1. Wheat seeds of uniform size were washed thrice with distilled water and germinated on wet filter papers in Petri dishes for germination at 20 ℃. After 2 days, nine germinating seeds of each accession were evenly planted in one pot, and each pot was thinned to six seedlings after 10 days of sowing. Each pot (height 15 cm; diameter 20 cm) was filled with 4.5 kg of soil (nutritional soil: sieved tillage soil, 1:1) to easily obtain the whole root system.

For OPC, the pots were randomly buried in a field on 15 October 2019 at Xinxiang (35.29° N, 113.83° E), Henan, China. To facilitate management, 10 blocks were set in the field, each containing approximately 40 pots. The plants were watered to 70–80% of the field capacity. The seedlings developed to the three-leaf stage after 25 days of sowing with a mean temperature of 14.85 °C and 0 mm of rainfall. For IPC, the pots were randomly placed in a phytotron. The seedlings were grown under 80% relative humidity, a 16 h light (20 °C) / 8 h darkness (18 °C) photoperiod, and 8000 Lux light intensity. The seedlings developed to the three-leaf stage after 30 days of sowing. The roots were cleaned with water. For each wheat accession under the two environments, six biological replications were sampled for further RSA trait measurement.

### 4.2. RSA Trait Measurement

The root samples were placed in plexiglass trays (200 mm × 250 mm) containing 4–6 mm of distilled water. The roots were manipulated to minimise tangling and overlap. Nine RSA traits (Table 6), TRL, TRA, ARD, TRV, NRT, RL, RA, RV, and RT, were measured using a recording scanner (Epson 1680, Suwa, Japan). The images were analysed using Win RHIZO Pro Vision 5.0a (Regent Instruments Inc., Quebec, QC, Canada), which was operationally semi-automated. 

### 4.3. Phenotypic Data Analysis for RSA Traits

SPSS (Version 17.0) and R packages (R Version 4.1.1) were used for data analysis. Genotype, environments, and genotype-by-environment interactions were considered as random effects in a linear mixed model (LMM_4 K). The lme4 package in the R 4.1.1 program was used to estimate the BLUPs. The *H*^2^ of the RSA traits was calculated using the mean values of each experiment among six replications using the formula:

H2=σG2σG2+σGE2n+σe2nr  

where $\sigma_{G}^{2}$ is the genetic variance, $\sigma_{GE}^{2}$ is the genotype × environment, $\sigma_{e}^{2}$ is the error variance, *n* represents the number of environments, and *r* represents the number of replications. The mean values were used to analyse the differences using a *t*-test. The CV was calculated for all the RSA traits [63].

### 4.4. Population Structure

Principal component analysis of the population was performed using the GCTA software [64]. For population structure analysis, a Bayesian model-based (Markov Chain Monte Carlo) clustering approach was used in the STRUCTURE software v2.3.4 with unlinked markers (*r*^2^ = 0) [65]. The *K*-value, representing the number of subgroups, was set from 2 to 20, and five independent runs for each *K* were performed with the burn-in length set to 20,000 and iterations set to 10,000. The most likely number of subpopulations was determined using the ∆*K* method based on the rate of change in LnP (D) between successive *K*-values [66].

### 4.5. LD Analysis

Genome-wide LD analysis for the A, B, and D genomes was performed using the PLINK software. The squared allele-frequency correlations (*r*^2^) were used to estimate the LD by applying pair-wise comparisons among the filtered SNP markers. The values for genomes A, B, and D were plotted against the genetic distance to determine LD decay. The parameters for calculating *r*^2^ were set to --r2 --ld-window-kb 30000 --ld-window 1000 --ld-window-r2 0. A locally weighted polynomial regression (LOESS) curve was drawn to fit the data using second-order local weighted scatter plot smoothing in the R program. The critical distance spanning the QTL was defined based on the intersection point of the fitted LOESS curve with the LD (*r*^2^ = 0.1). 

### 4.6. Genotyping Data Using 660K SNp Chip

The wheat panel was genotyped using the Affymetrix Wheat 660K SNP Chip [48], which was provided by the Beijing CapitalBio Technology Company (http://www.capitalbiotech.com, accessed on 8 January 2022). SNP genotyping calling and allele clustering were performed using the polyploid version of the Affymetrix Genotyping Console software. To ensure genotyping accuracy, quality control, and filtering of raw data with missing data > 10%, a minor allele frequency < 5%, or Hardy-Weinberg Equilibrium > 0.01 were performed. The remaining high-quality SNP markers were used for GWAS. The sequence of each polymorphic SNP was identified using Basic Local Alignment Search Tool (BLAST)n 2.2.29+, and the physical positions of all SNPs were determined by blasting them with the wheat reference genome IWGSC RefSeq v1.0. The parameters of the blast result were set to: ratio (AL/QL) ≥ 0.95, identity ≥ 0.95, and E-value < 1 × e^−10^. Two genetic diversity parameters, PIC and *He*, were calculated for each SNP marker and each chromosome using the formulas described by Botsteinet et al. [67] and Nei [68], respectively.

### 4.7. GWAS

GWAS was conducted using a univariate linear mixed model in the GEMMA software [69]. The suggestive threshold for *p*-value (*p* = 1/*Ne)* was calculated using a modified Bonferroni correction (genetic type 1 Error calculator, version 0.2), where *Ne* represents the effective number of SNP markers [70]. A threshold was established at −log_10_*p* ≥ 4 to determine the marker–trait association significance. Moreover, the *p*-value of −log_10_*p* ≥ 3.5 was used to establish a second, less restrictive threshold. Significant markers from GWAS were visualised in Manhattan plots, and important *p*-value distributions were visualised using quantile-quantile (Q-Q) plots drawn with the *qqman* package in R 4.1.1. The phenotypic variance (*R*^2^) explained by significant SNPs was evaluated using the GCTA software [64]. 

### 4.8. Candidate Gene Identification, RNA Extraction, and RT-qPCR Verification

Candidate genes were selected using functional annotation and haplotype analysis. For haplotype analysis, the significant SNPs in QTL clusters were used to identify non-synonymous polymorphisms. The phenotypic differences between the haplotypes of the nine RSA traits were analysed to identify the candidate genes.

The root, stem, and leaf tissues were sampled from wheat plants of cv. Chinese Spring grown in the field at the jointing and heading stages, respectively. The total RNA was extracted using the TRIzol reagent (TaKaRa, Ohtsu, Japan) according to the manufacturer’s instructions. One microgram of the total RNA was used for first-strand cDNA synthesis using the HiScript III First-Strand cDNA Synthesis Kit (Vazyme, Nanjing, China). qPCR was performed on an ABI 7500 real-time PCR system (Applied Biosystems, Forest City, CA, USA) using SYBR Premix Ex Taq Kit (TaKaRa, Japan). Three biological replications were used for each experiment. *TaActin3* was used as an internal control [71], and the relative expression value was calculated using the 2^−∆∆Ct^ method. All primers used for RT-qPCR were listed in Table S6.

## Figures and Tables

**Table 1:** Components of eight subpopulations.

Sub-Population	Number of Accession	Exotic Variety	Local Landrace	Local Cultivar
Sp1	66	11	6	49
Sp2	41	7	5	29
Sp3	64	5	1	58
Sp4	47	13	15	19
Sp5	41	10	3	28
Sp6	33	0	0	33
Sp7	30	1	1	28
Sp8	66	19	7	40

**Table 2:** Phenotypic analysis of nine RSA traits for this wheat panel under two environments.

Trait	E	Mean	SD	Min	Max	bk	bs	CV (%)	*H*^2^ (%)	*p*-Value	<0.001
TRL	OPC	236.06	94.96	64.25	645.14	1.84	1.14	40.22%	77.83%	7.96175 × 10^−51^	***
	IPC	147.91	50.03	53.91	336.76	0.65	0.83	33.82%			
TRA	OPC	31.68	12.98	9.50	86.52	1.25	0.91	40.97%	76.86%	2.91719 × 10^−81^	***
	IPC	16.45	4.93	7.11	36.00	0.55	0.80	29.94%			
ARD	OPC	0.43	0.07	0.27	0.58	−1.06	−0.32	16.91%	66.78%	9.8363 × 10^−58^	***
	IPC	0.36	0.03	0.28	0.45	−0.29	0.37	8.52%			
TRV	OPC	0.35	0.17	0.07	1.09	1.51	0.95	47.94%	76.86%	4.92408 × 10^−89^	***
	IPC	0.15	0.04	0.07	0.44	6.18	1.60	29.81%			
NRT	OPC	360.80	186.97	113.00	1703.67	8.55	2.20	51.82%	72.38%	1.24864 × 10^−28^	***
	IPC	233.30	110.08	78.67	699.83	2.14	1.36	47.18%			
RL	OPC	113.91	63.29	18.27	429.16	4.95	1.83	55.56%	75.71%	3.14966 × 10^−23^	***
	IPC	75.99	35.82	19.24	225.26	1.06	0.98	47.14%			
RA	OPC	6.90	3.59	1.15	24.26	3.78	1.62	52.08%	76.15%	6.77309 × 10^−28^	***
	IPC	4.48	2.11	1.01	11.82	0.44	0.89	47.13%			
RV	OPC	0.04	0.02	0.0067	0.1214	3.04	1.49	50.12%	76.06%	7.24198 × 10^−31^	***
	IPC	0.02	0.01	0.0053	0.0626	0.25	0.87	48.38%			
RT	OPC	311.12	187.64	45.33	1641.83	8.30	2.20	60.31%	71.15%	4.80007 × 10^−17^	***
	IPC	216.96	106.31	67.33	678.00	2.22	1.36	49.00%			

E, Environments; bk, kurtosis; bs, skewness; CV, coefficient of variation; *H*^2^, heritability; *** Significant at *p* ≤ 0.001.

**Table 3:** Summary of the genetic diversity in three sub-genomes and chromosomes of this wheat panel and evaluation of the effective number of independent SNPs, including the suggested *p*-value thresholds.

Chromosome	No. of Markers	Effective Number	Effective Ratio	Suggested *p*-Value	% Markers	Length (Mb)	Marker Density	*He*	PIC
1A	28793	5643.19	0.20	1.77 × 10^−4^	7.00	594.02	48.47	0.65	0.26
2A	28676	6088.17	0.21	1.64 × 10^−4^	6.97	780.76	36.73	0.71	0.28
3A	19366	4607.43	0.24	2.17 × 10^−4^	4.70	750.73	25.80	0.68	0.28
4A	17856	4241.96	0.24	2.36 × 10^−4^	4.34	744.54	23.98	0.66	0.27
5A	22482	4935.88	0.22	2.03 × 10^−4^	5.46	709.76	31.68	0.78	0.31
6A	16438	3851.64	0.23	2.60 × 10^−4^	3.99	617.97	26.60	0.73	0.29
7A	28190	6297.36	0.22	1.59 × 10^−4^	6.85	736.69	38.27	0.68	0.27
1B	20584	4750.1	0.23	2.11 × 10^−4^	5.00	689.38	29.86	0.75	0.30
2B	28715	6701.21	0.23	1.49 × 10^−4^	6.98	801.25	35.84	0.73	0.29
3B	45766	7920.52	0.17	1.26 × 10^−4^	11.12	830.70	55.09	0.78	0.31
4B	13130	3016.41	0.23	3.32 × 10^−4^	3.19	673.47	19.50	0.72	0.29
5B	33874	6596.79	0.19	1.52 × 10^−4^	8.23	713.02	47.51	0.79	0.31
6B	25549	5554.71	0.22	1.80 × 10^−4^	6.21	720.95	35.44	0.69	0.28
7B	17040	4491.9	0.26	2.23 × 10^−4^	4.14	750.61	22.70	0.72	0.29
1D	10597	3058.85	0.29	3.27 × 10^−4^	2.57	495.44	21.39	0.68	0.28
2D	10430	3731.57	0.36	2.68 × 10^−4^	2.53	651.81	16.00	0.69	0.28
3D	7291	2668.7	0.37	3.75 × 10^−4^	1.77	615.48	11.85	0.67	0.27
4D	4128	1702.71	0.41	5.87 × 10^−4^	1.00	509.85	8.10	0.66	0.27
5D	8437	3285.78	0.39	3.04 × 10^−4^	2.05	566.04	14.91	0.65	0.26
6D	7486	2940.59	0.39	3.40 × 10^−4^	1.82	473.56	15.81	0.64	0.26
7D	10110	3669.4	0.36	2.73 × 10^−4^	2.46	638.65	15.83	0.64	0.26
A genome	161801				39.31	4934.47	32.79	0.70	0.28
B genome	184658				44.86	5719.38	32.29	0.74	0.30
D genome	58479				14.21	3950.83	14.80	0.66	0.27
Total	404938	95754.87				14,064.68	28.79	0.70	0.28
Average				2.51 × 10^−4^					

**Table 4:** QTLs and QTL clusters with RSA traits and overlapping with QTLs from previous studies.

Traits	SNP	CHR	Mb	*p*	−log_10_ (*p*)	R^2^ (%)	Reference
ARD	AX-94798162	2B	245.89	8.53 × 10^−5^	4.07	1.23	
ARD	AX-94757865	2B	797.54	6.71 × 10^−5^	4.17–4.38	2.24–2.76	
ARD	AX-110887844	2D	380.98	7.38 × 10^−5^	4.13	2.57	
ARD	AX-109125375	2D	640.04	9.95 × 10^−5^	4.00–5.38	1.82–3.41	
TRV	AX-108892435	3A	21.28	7.86 × 10^−5^	4.10	2.58	
ARD	AX-111490934	3B	49.29	9.67 × 10^−5^	4.01	3.59	
TRV	AX-94897906	4A	102.39	9.21 × 10^−5^	4.04	2.28	
TRV	AX-110712645	4B	606.85	7.29 × 10^−5^	4.14–4.74	4.77–5.11	
TRV	AX-95073736	5A	632.60	5.59 × 10^−5^	4.25	2.64	
TRL	AX-111537095	5B	639.68	9.81 × 10^−5^	4.01–4.30	4.16–4.95	
ARD	AX-95093123	5D	385.39	2.69 × 10^−5^	4.57	2.31	
ARD	AX-94999582	6A	104.36	6.99 × 10^−5^	4.16	4.23	
ARD	AX-94827303	6A	115.18	8.43 × 10^−5^	4.07	2.84	
ARD	AX-94502864	6B	710.32	7.22 × 10^−6^	5.14	3.17	[27]
ARD	AX-94826824	6D	60.14	5.84 × 10^−5^	4.23	2.36	
ARD	AX-94468039	6D	86.37	7.64 × 10^−5^	4.12	3.18	
ARD	AX-110837768	6D	464.84	6.32 × 10^−5^	4.20	2.29	[28]
ARD	AX-94849869	7A	19.01	7.99 × 10^−5^	4.10	3.46	
RT	AX-108852993	7A	40.26	7.97 × 10^−5^	4.10	3.76	
NRT	AX-94970901	7A	692.05	8.53 × 10^−5^	4.07	4.03	[27]
RT	AX-95181992	7B	228.81	5.95 × 10^−5^	4.23	3.84	
ARD	AX-109581543	7B	403.54	3.66 × 10^−5^	4.44	3.03	[29]
ARD	AX-110360435	7D	104.09	9.83 × 10^−5^	4.01	2.24	
TRA/TRV	AX-111630548	2A	61.28	8.82 × 10^−5^	4.05–4.29	2.68–3.20	
TRA/TRV	AX-110564036	2A	740.01	8.78 × 10^−5^	4.06–4.11	3.41–3.69	
TRA/TRV	AX-109537244	2B	694.26	8.70 × 10^−5^	4.06–4.29	2.52–2.64	
NRT/RT	AX-95193648	3A	0.56	2.39 × 10^−5^	4.62–4.92	3.97–4.29	
NRT/RT	AX-94711043	3B	0.99	1.17 × 10^−5^	4.93–5.14	4.28–4.42	
NRT/RT	AX-109337192	3B	2.28	3.94 × 10^−5^	4.40–4.80	3.77–4.16	
TRL/RL/RA/RV	AX-109237510	3D	549.74	9.90 × 10^−5^	4.00–4.16	3.68–4.58	
NRT/RT	AX-110444857	4A	584.71	9.92 × 10^−5^	4.00–4.37	4.64–4.78	
NRT/RT/RL/RA/RV	AX-86179446	5B	571.23	9.98 × 10^−5^	4.00–5.73	3.99–6.77	[28]
NRT/RT	AX-110522657	5D	464.07	8.90 × 10^−5^	4.05–4.66	3.97–5.60	
NRT/RT	AX-111060833	6B	127.23	5.66 × 10^−5^	4.25–4.42	4.00–4.15	
TRL/TRA	AX-95155829	7B	36.49	4.72 × 10^−5^	4.33–4.58	4.51–4.80	
TRL/TRA	AX-94759416	7B	594.41	8.96 × 10^−5^	4.05–4.23	4.00–5.28	

**Table 5:** Published studies on RSA traits and their chromosome localisations.

Chr	Traits	Reference
1A	RGA, TRL, NRT, TRV, RHL, MRL, TRA, ARD	[5,22,23,26,29,30,32,34,35,44,45]
1B	TRL, NRT, RHL, TRA, ARD, TRV, MRL	[5,22,23,26,27,29,32,34,35,36,45]
1D	NRT, TRA, TRL, ARD, NRT, TRA	[28,30,32,37]
2A	TRL, TRV, ARD, TRA, NRT, RGA, MRL	[5,23,30,32,33,34,35,36,37,38,44]
2B	RGA, TRL, TRV, TRA, ARD, NRT, MRL	[5,19,22,23,24,29,30,31,32,33,34,35,37,38,44,45]
2D	TRL, RHL, TRV, TRA, ARD, NRT, MRL	[22,24,26,31,32,33,37,45]
3A	RGA, TRL, NRT, RHL, MRL, TRV, TRA, ARD	[5,22,23,24,26,30,32,33,34,35,36,43,45]
3B	RGA, TRL, NRT, MRL, ARD, TRV, TRA	[5,22,23,24,30,33,34,35,38,39,45]
3D	ARD, TRL, NRT	[32,38,39,45]
4A	RGA, TRL, NRT, TRV, TRA, ARD	[22,23,25,28,30,32,33,34,35,37,38,42,45]
4B	TRL, NRT, ARD, TRA, RGA, TRV	[5,22,23,28,30,31,32,34,35,36,37,39,42,43,45]
4D	TRL, TRA, NRT, MRL, TRV	[31,33,34,38]
5A	TRL, ARD, TRV, NRT, TRA, MRL	[5,19,22,23,25,30,33,34,35,38,45]
5B	RGA, TRL, NRT, TRA, MRL, TRV, TRA, ARD	[22,23,28,29,30,31,32,33,34,35,45,56,58]
5D	TRV, TRL, TRA, ARD, MRL	[22,28,29,32,34,37,45]
6A	RGA, NRT, MRL, TRL, ARD, TRA	[5,22,23,29,31,34,35,36,44]
6B	RGA, TRL, NRT, MRL, RHL, TRA, ARD, TRV	[5,19,22,23,24,26,29,34,35,36,39,43]
6D	NRT, TRL, TRA, ARD, NRT, MRL, RHL	[24,26,28,29,33,34,40,46]
7A	RGA, TRL, NRT, TRA, MRL, ARD, TRV	[5,22,23,24,29,30,31,32,33,34,35,37,39,40,41,42,45]
7B	RGA, NRT, TRL, TRA, MRL, ARD, RHL, TRV	[5,19,22,23,24,26,29,30,31,33,34,35,36,39,45]
7D	NRT, TRA, TRL, TRV, MRL	[22,29,30,32,34,37,38,40,45]

**Table 6:** Abbreviations for RSA traits and their units.

Acronym	RSA Traits	Units
TRL	Total root length	Centimetres (cm)
TRA	Total root surface area	Square centimetres (cm^2^)
ARD	Average root diameter	Millimetres (mm)
TRV	Total root volume	Cubic centimetres (cm^3^)
NRT	Number of root tips	Number (no.)
RL	Root length (root diameter ≤ 0.3 mm)	Centimetres (cm)
RA	Root surface area (root diameter ≤ 0.3 mm)	Square centimetres (cm^2^)
RV	Root volume (root diameter ≤ 0.3 mm)	Cubic centimetres (cm^3^)
RT	Root tips (root diameter ≤ 0.3 mm)	Number (no.)

## Data Availability

Not applicable.

## Supplementary Materials

The following supporting information can be downloaded at: https://www.mdpi.com/article/10.3390/ijms23031843/s1.
