# Supplementary material for: Genome-Wide Association Study on Root System Architecture and Identification of Candidate Genes in Wheat (Triticum aestivum L.)
Source: Int J Mol Sci. 2022 Feb 6;23(3):1843. doi: 10.3390/ijms23031843 (PMC8836572; doi:10.3390/ijms23031843)
Supplement: Supplementary file 1 [file ijms-23-01843-s001.zip › Supplementary Files/Figure S2 Genome-wide association study for RSA traits.pdf]

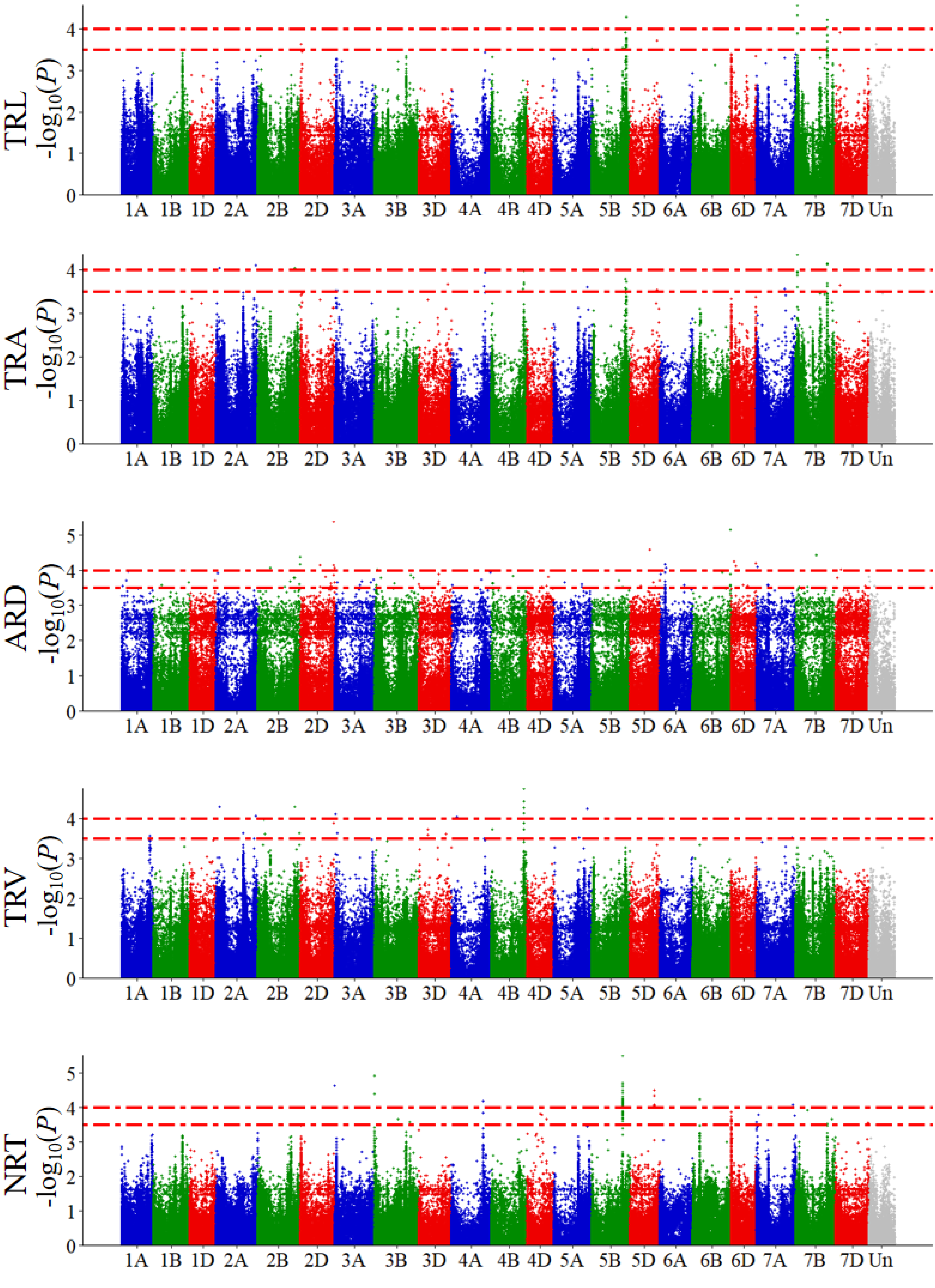
TRL
TRA
ARD
TRV
NRT
$-\log_{10}(P)$
1A 1B 1D 2A 2B 2D 3A 3B 3D 4A 4B 4D 5A 5B 5D 6A 6B 6D 7A 7B 7D Un

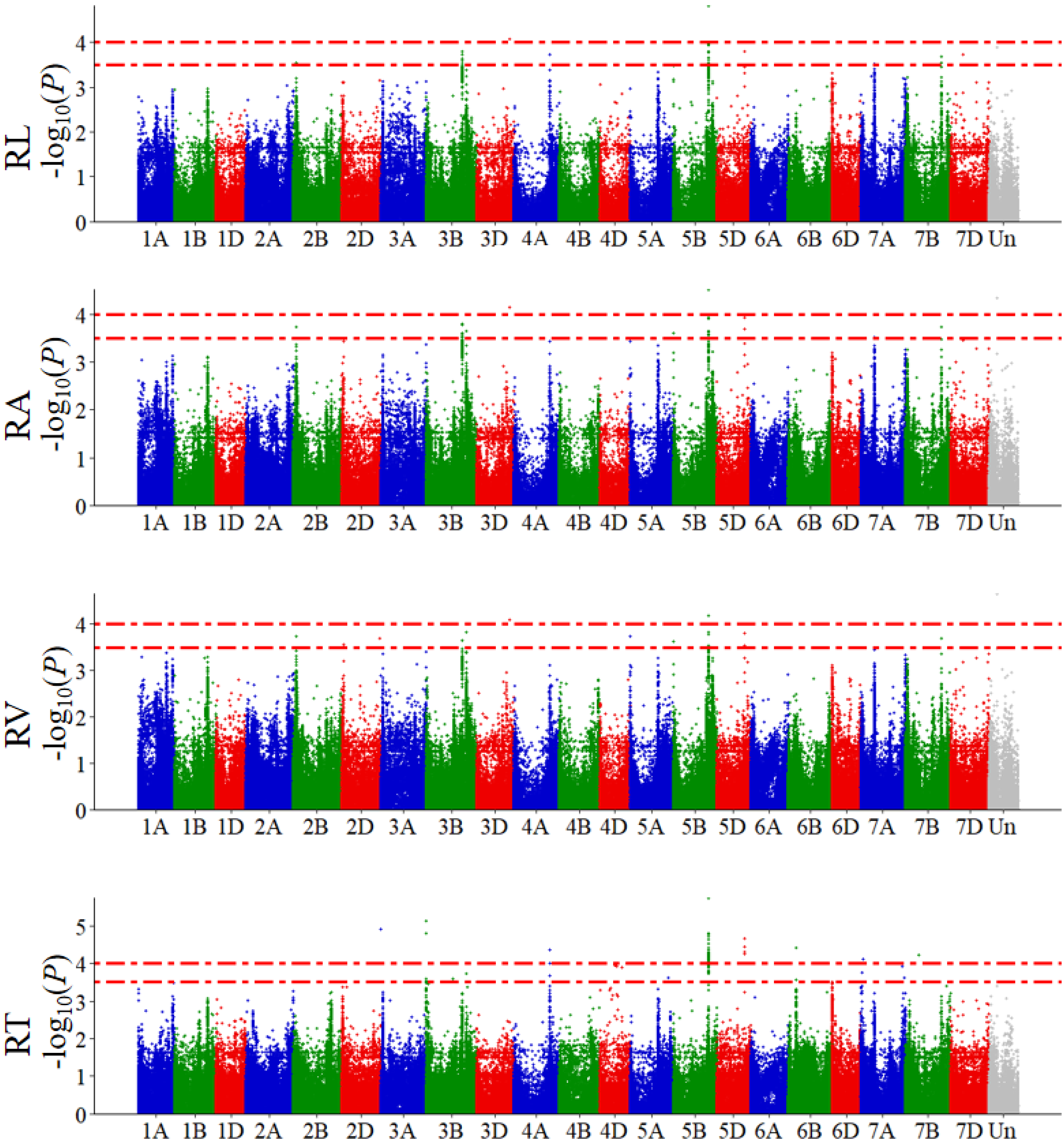

Figure S2. Genome-wide association study for RSA traits.
The dashed horizontal lines in red show the genome-wide significance threshold $-\log_{10}(P\text{-value})$ of 3.5 and 4. The A, B and D sub-genomes are indicated in blue, green and red, respectively.
