# Supplementary figures and images for: Genome-Wide Association Study on Root System Architecture and Identification of Candidate Genes in Wheat (Triticum aestivum L.)
Source: Int J Mol Sci. 2022 Feb 6;23(3):1843. doi: 10.3390/ijms23031843 (PMC8836572; doi:10.3390/ijms23031843)

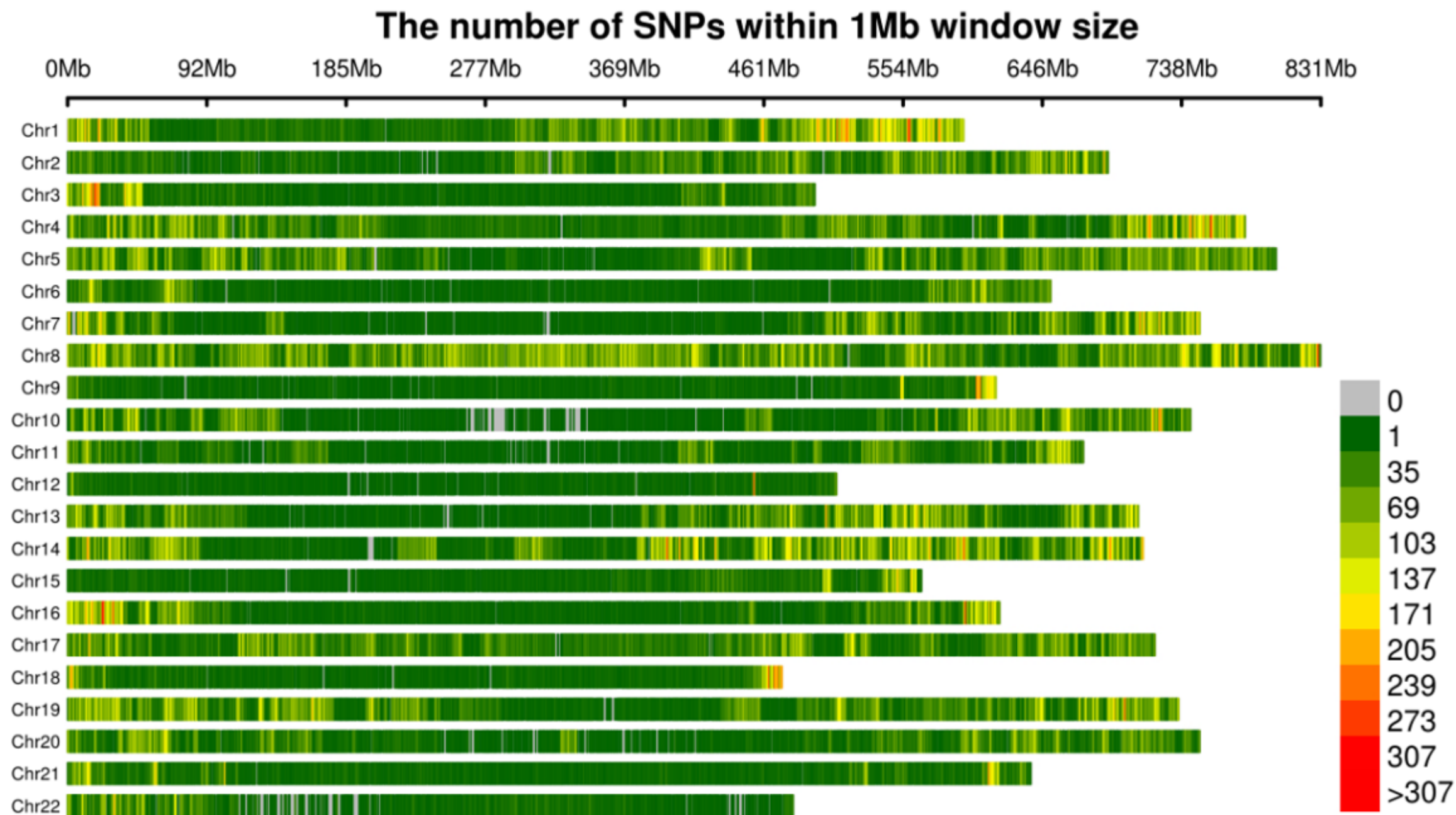

**Figure S1.** The number of SNPs within 1Mb window size.

Supplement: Supplementary file 1 [file ijms-23-01843-s001.zip › Supplementary Files/Figure S1 The number of SNPs within 1Mb window size.pdf]
